# Missense mutations in Desmocollin-2 N-terminus, associated with arrhythmogenic right ventricular cardiomyopathy, affect intracellular localization of desmocollin-2 in vitro

**DOI:** 10.1186/1471-2350-8-65

**Published:** 2007-10-26

**Authors:** Giorgia Beffagna, Marzia De Bortoli, Andrea Nava, Michela Salamon, Alessandra Lorenzon, Manuela Zaccolo, Luisa Mancuso, Luca Sigalotti, Barbara Bauce, Gianluca Occhi, Cristina Basso, Gerolamo Lanfranchi, Jeffrey A Towbin, Gaetano Thiene, Gian Antonio Danieli, Alessandra Rampazzo

**Affiliations:** 1 Department of Biology, University of Padua, Padua, Italy; 2 Department of Cardiothoracic-Vascular Sciences, University of Padua Medical School, Padua, Italy; 3 Venetian Institute of Molecular Medicine, Padua, Italy; 4 Cancer Bioimmunotherapy Unit, Department of Medical Oncology, Centro di Riferimento Oncologico, Istituto di Ricovero e Cura a Carattere Scientifico, Aviano, Italy; 5 Institute of Pathology, University of Padua, Padua, Italy; 6 CRIBI Biotecnology Centre, University of Padua, Padua, Italy; 7 Department of Pediatrics, Section of Cardiology, Baylor College of Medicine, Houston, Texas, USA

## Abstract

**Background:**

Mutations in genes encoding desmosomal proteins have been reported to cause arrhythmogenic right ventricular cardiomyopathy (ARVC), an autosomal dominant disease characterised by progressive myocardial atrophy with fibro-fatty replacement.

We screened 54 ARVC probands for mutations in desmocollin-2 (*DSC2*), the only desmocollin isoform expressed in cardiac tissue.

**Methods:**

Mutation screening was performed by denaturing high-performance liquid chromatography and direct sequencing.

To evaluate the pathogenic potentials of the *DSC2 *mutations detected in patients affected with ARVC, full-length wild-type and mutated cDNAs were cloned in eukaryotic expression vectors to obtain a fusion protein with green fluorescence protein (GFP); constructs were transfected in neonatal rat cardiomyocytes and in HL-1 cells.

**Results:**

We identified two heterozygous mutations (c.304G>A (p.E102K) and c.1034T>C (p.I345T)) in two probands and in four family members. The two mutations p.E102K and p.I345T map to the N-terminal region, relevant to adhesive interactions.

In vitro functional studies demonstrated that, unlike wild-type DSC2, the two N-terminal mutants are predominantly localised in the cytoplasm.

**Conclusion:**

The two missense mutations in the N-terminal domain affect the normal localisation of DSC2, thus suggesting the potential pathogenic effect of the reported mutations. Identification of additional DSC2 mutations associated with ARVC may result in increased diagnostic accuracy with implications for genetic counseling.

## Background

Arrhythmogenic right ventricular cardiomyopathy (ARVC) (MIM #107970) is a myocardial disease in which myocardium of the right ventricular free wall is partially or almost entirely replaced by fibro-fatty tissue [1], leading to structural and functional abnormalities of the right ventricle, electrocardiographic depolarization/repolarization changes and arrhythmias of right ventricular origin [2]. Clinical symptoms are manifested in the early adulthood.

The disease is transmitted as an autosomal dominant trait with reduced penetrance. Although pathogenic mutations underlying ARVC were detected in *RyR2 *(cardiac ryanodine receptor) [3] and in *TGFβ3 *(transforming growth factor-β3) [4] genes, finding mutations in genes encoding desmosomal proteins, namely Desmoplakin (*DSP*), Plakophilin-2 (*PKP2*) and Desmoglein-2 (*DSG2*) [5-7] led to current idea that ARVC is due to desmosomal dysfunction [8]. This was further strengthened by two recent studies that reported mutations in the desmosomal desmocollin-2 (*DSC2*) gene as the cause of ARVC [9,10]. Actually, also the autosomal recessive form of ARVC, known as Naxos syndrome, associated with palmoplantar keratoderma and peculiar woolly hairs (MIM #601214) is due to mutation in the desmosomal protein Plakoglobin (JUP) [11]. Moreover, intercalated discs ultrastructural abnormalities consisting of decreased desmosome number and intercellular gap widening were observed in ARVC biopsy samples [12].

Desmosomes anchor stress-bearing intermediate filaments at sites of strong intercellular adhesion; the resulting scaffold plays a key role in providing mechanical integrity of tissues which undergo high mechanical stress, such as epidermis and heart [13]. Desmosomes include proteins from at least three distinct gene families: cadherins, armadillo proteins and plakins [14]. Desmosomal cadherins include DSGs and DSCs; members of both subfamilies are single-pass transmembrane glycoproteins, mediating Ca^2+^-dependent cell-cell adhesion.

We report here two novel mutations of the *DSC2 *gene detected in patients affected with ARVC and their functional characterization.

## Methods

### Clinical evaluation

Clinical diagnosis of ARVC was based on major and minor criteria established by the European Society of Cardiology/International Society and Federation of Cardiology Task Force [15]. The study involved a cohort of 54 patients of Italian descent. Each patient underwent cardiological examination. The clinical study protocol included 12-lead electrocardiogram (ECG), signal-averaged ECG (SAECG), 24-hour Holter ECG, and 2-dimensional echocardiography, performed according to previously reported methods [2]. All clinical investigations and blood sampling for DNA analysis were performed under informed consent, according to the pertinent Italian legislation and in compliance with Helsinki declaration.

### Mutation screening

Fifty-four Italian probands (33 males and 21 females), which proved negative for mutations of *DSP*, *PKP2*, *DSG2 *and *TGFβ3 *genes, were screened for *DSC2 *mutations by denaturing high-performance liquid chromatography (DHPLC) and direct sequencing.

Primers flanking each exon of the human *DSC2 *gene were designed by PRIMER3. PCR amplifications were performed in a final volume of 25 μl, containing 50 ng of genomic DNA, 400 nmol/l of each primer (Sigma Genosys), 100 μmol/l of each dNTP, 1.5 μmol/l MgCl_2_, and 0.8 U of Taq DNA Polymerase (TaqGold, Applied Biosystems). PCR primers and DHPLC conditions are reported in Table 1. DHPLC analysis was performed with the use of WAVE Nucleic Acid Fragment Analysis System 3500 HT (Transgenomic Ltd NE, USA). Samples showing a change in DHPLC pattern were directly sequenced. *DSC2 *sequences were compared to reference sequence NM_024422.

**Table 1:** PCR primers and conditions for DHPLC analysis of *DSC2 *gene

**EXON**	**bp**	**T°C DHPLC**	**Forward 5' 3'**	**Reverse 5' 3'**
1+5'UTR	708	Direct sequencing	TCAGACCTCGCTCTGTAATTGA	TATCCCCGTTCCCCTAGTTT
2	199	Direct sequencing	ACACATTAAAGTTTTCTTTTTAT	GGCGTATATGTACCACAGCA
3	400	54,8/55,4	CCCCACGTGCATACATTACT	TGGTTTTCATTCGTCTTTAAGC
4	269	55,6/58.5	CCCCTACCCAGCTAATCCTC	GGAAACTATAGACTCCCACAGCA
5	332	56,5/57,2	TGAAAGCTCTGCTGAAATAAAGA	GGAGTAGCCAGAGCATTGGT
6	266	54.7	GCCAAAATGAATTTGAAGCATAC	TTGAAACACAGTTAATTTGCCATA
7	384	55/58/59,5	CATAGAACATGTGAATGTTTTGGA	CAAAACCAGCATACTCCAAGG
8	252	54,3/55.6	GTTGGTGCTTTCCCCCAATA	AGGCCAGAGATGTGCATATTA
9	324	52.2/53.5/55.3	CATCGTGTTCAATTTTTGTGA	CCTTTCTTTCCATTAAATTCTAGC
10	374	56,5/57,9	ACTCGTTAGCATTGCCAAAT	TAACGTAACAAAATAAGCTA
11	375	51,7/56	CAAGAAGTAGCAGTGGCATAAGG	AACAGAGTGCATGTATCCAGCTT
12	345	57/58/59	GTGTTCAGTGCATACTTTTGTGG	GCAGACATCCTGATGTTGAAAA
13	356	57,2/58	TGTTCAGAAGAAATCAGTGACA	GTGTCTTGAAAGTTACTTTAAAGG
14	263	57.7	GATTTATGTGTGTATTAACCATTG	CGCATTATAAGCGAATTCATCC
15	a 194	56.2/60.6/61.8	CATAATTTTGTGTTCCTCTCTGT	AGGATTCCGAGGTCTGGTGT
	b 332	61/61,4/62,2	GGCTTCACAACCCAAACTGT	TGAAAATTATAGTCAGAATCCAGT
15'	226	53,7/55,2	GCCACATGCGTGACTTTTAG	ACTTTCTGCCAAGGGGAAAA
16	397	56,3/56,8	CAATGAAAGGTAAATCAAAGCAA	AAAAACCCCCACAAATAGCA

A control group of 250 healthy and unrelated subjects (500 chromosomes) from Italian population was used for assessing possible polymorphism of detected DNA variants. All controls were matched to the probands by ancestry.

Presence of the missense mutations p.E102K and p.I345T was confirmed by restriction digest of PCR amplicons with *TspR1 *restriction enzyme (NEW ENGLAND BioLabs).

### Cell cultures

Cardiac ventricular myocytes from one to three days old Crl:(WI)BR-Wistar rats (Charles River Laboratories, Wilmington, MA) were prepared as previously described [16]. All animal procedures were followed according to institutional guidelines.

HL-1 cardiac myocytes were maintained as previously reported [17].

### Plasmid construction

The cDNA of human *DSC2a *was kindly provided by Dr W.W. Franke (Heidelberg, Germany) and human *DSC2a *full-length coding sequence (GenBank NM_024422) was PCR-amplified with the following primers: *DSC2a*-clonF: ATTATGGAGGCAGCCCGCCC; *DSC2a*-clonR: GTCTCTTCATGCATGCTTCTGCTAG. The resulting fragment was cloned into pcDNA3.1/CT-GFP-topo eukaryotic expression vector (Invitrogen) which contains cDNA coding for green fluorescent protein (GFP), and verified by sequence analysis.

### Site directed mutagenesis

Site-directed mutagenesis was performed on *DSC2a*-pcDNA3.1/CT-GFP, in order to reproduce two *DSC2 *naturally occurring mutations: p.E102K (EF017811), and p.I345T (EF017812). We used the QuikChange XL Site-Directed Mutagenesis Kit (Stratagene) and the following mutagenic primers: E102K-F: TACCATATTACTTTCCAACACTAAGAACCAAGAAAAGAAGAAAAT; E102K-R: ATTTTCTTCTTTTCTTGGTTCTTAGTGTTGGAAAGTAATATGGTA; I345T-F: CAACTTCAACTTGTATCATTAACACTGATGATGTAAATGACCACTTGC; I345T-R: GCAAGTGGTCATTTACATCATCAGTGTTAATGATACAAGTTGAAGTTG. All clones were verified by sequence analysis.

### Transfection of neonatal rat cardiomyocytes and HL-1 cells with wild-type and mutant DSC2a-pcDNA3.1/CT

After 1 day in culture, cardiomyocytes were transfected with 3 μg of plasmid DNA per well and 8 μl of transfection reagent (transfectin lipid reagent Biorad). After 24 hours of transfection, cells were fixed for 20 minutes with 4% paraformaldehyde in phosphate-buffered saline, and then permeabilized with 0.1% Triton X-100 for 10 minutes. HL-1 cells were transfected with 0.8 μg of plasmid DNA per plate, 6.4 μl of enhancer reagent, and 8 μl of effectene reagent (Qiagen). At 48 hours post-transfection, HL-1 cells were fixed with cold methanol/acetone (1:1) for 20 minutes at -20°C. Each set of subsequent experiments was performed in triplicate in 24 mm glass coverslips and was repeated 3 times.

### Immunostaining and confocal imaging on neonatal rat cardiomyocytes and HL-1 cells

Neonatal rat cardiomyocytes were stained with anti-alpha actinin antibody (Sigma) for two hours at room temperature, washed three times with PBS and incubated with Alexa fluor 543-conjugated antimouse antibody (Molecular Probes, Eugene, Ore). HL-1 cells were incubated with the murine mAb (clone DG3.10) against bovine dsg (PROGEN, Heidelberg, Germany), which reacts with a carboxy terminal epitope of desmoglein for 30 min at 37°C, washed three times with PBS, and incubated with TRITC conjugated antibodies (DAKO).

Slides were inspected and photographed using a Radiance 2000 confocal microscope (BioRad) with a 60× oil objective.

## Results

### Genetic analysis

**Figure 1:**
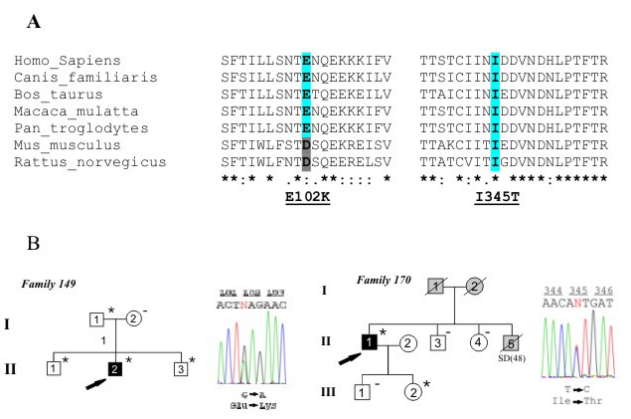
a) Evolutionary conservation of the *DSC2 *missense mutations p.E102K and p.I345T among 7 species: *H. sapiens *(AAH63291.1), *C. familiaris *(CAA05309.1), predicted *B. taurus *(XP_615164.2), predicted *M. mulatta *(XP_001102096.1), predicted *P. troglodytes *(XP_512077.2), *M. musculus *(AAH57867.1) and *R. norvegicus *(NP_001028860.1). The mutated amino acids are coloured, and the identities across species are indicated by a different background. b) Pedigrees of ARVC probands carrying *DSC2 *mutations. Black, white, and grey symbols represent clinically affected individuals, unaffected individuals, and individuals of unknown disease status, respectively. SD indicates sudden death. Presence (*) or absence (-) of the *DSC2 *mutation is indicated. Arrows indicate index cases. Sequence electropherograms, on the right side of the pedigrees, show the two *DSC2 *missense mutations (c.304G>A (p.E102K) and c.1034T>C (p.I345T)). Numbering of the nucleotides starts at ATG and refers to GenBank Accession number NM_024422.

Exon-by-exon analysis of coding sequences of *DSC2 *gene was performed on genomic DNA of 54 ARVC probands negative for mutation screening of genes known to be associated with ARVC. Two heterozygous point substitutions c.304G>A and c.1034T>C were detected in two patients (Figure 1). None of the detected nucleotide changes was found in a control group of 250 healthy and unrelated subjects (500 control chromosomes) from Italian population. Mutations were confirmed by restriction digest.

Variations c.304G>A and c.1034T>C result in predicted p.E102K and p.I345T amino acid substitutions. The mutated amino acids had completely different physico-chemical properties when compared to the wild type. Mutation p.E102K replaced a negatively-charged residue by a positively-charged one, whereas mutation p.I345T replaced a non polar hydrophobic amino acid by a polar hydrophilic amino acid.

Mutation p.E102K is located in the propeptide domain, whereas mutation p.I345T is located in the second extracellular cadherin (EC2) domain and altered the third amino acid of the EC domain consensus sequence [L/I/V-x-L/I/V-x-D-x-N-D-N/H-x-P] [18]. Cadherin domains are important for adhesive interactions and form Ca^2+ ^dependent rodlike structures.

Both these changes occurred in a residue highly conserved among species (Figure 1a), although in mouse and rat dsc2 protein, E102 is replaced by aspartic acid. However, these two amino acids show very similar physico-chemical properties.

### Clinical findings

Main clinical findings of patients carrying *DSC2 *mutations are reported in Table 2.

**Table 2:** Clinical and genetic findings in DSC2 mutation carriers

**Proband# (Family #)**	**Sex**	**Age at diagnosis/Last follow-up**	**Family history**		**12-lead ECG**				**SAECG**	**Arrhythmias**			**RV size/function**		**LV involv**	**Diagnostic criteria**	**Nucleotide change**	**AA change**	**GenBank accession number**
			Major	Minor	Negative T waves preocordial leads	Negative T waves inferior leads	IncomRBBB	Epsilon wave		PVCs >1000/24 h NSVT	LBBB SVT	VF	Major	Minor					

**I1 **(Fam149)	M	58	-	+	-	-	-	-	-	-	-	-	-	-	-	1 m	c.304G>A	p.E102K	EF017811
**II1 **(Fam149)	M	22	-	+	-	-	+	-	-	-	-	-	-	+	-	3 m	c.304G>A	p.E102K	EF017811
**II2 **(Fam149)	M	19	-	-	-	-	+	-	+	+	-	-	+	-	+	1 M/3 m	c.304G>A	p.E102K	EF017811
**II3 **(Fam149)	M	25	-	+	-	-	-	-	-	-	-	-	-	+	-	2 m	c.304G>A	p.E102K	EF017811
**II1 **(Fam170)	M	50	-	+	V4–V6	-	-	+	+	-	+	-	+	-	+	2 M/3 m	c.1034T>C	p.I345T	EF017812
**III2 **(Fam170)	F	15	-	+	-	-	-	-	-	-	-	-	-	+	-	2 m	c.1034T>C	p.I345T	EF017812

LBBB indicates left bundle-branch block; LV, left ventricular; NSVT, nonsustained ventricular tachycardia; RV, right ventricular; SVT, sustained ventricular tachycardia; VF, ventricular fibrillation; and M, major diagnostic criteria, m, minor diagnostic criteria

### p.E102K

The proband (II,2, Fam #149) (Figure 1b) was examined at the age of 19 years due to premature ventricular beats with left bundle branch block morphology. The ECG showed the presence of incomplete right bundle branch block, whereas late potentials were present at 40–80 filter setting. Two-D echocardiogram demonstrated a dilated right ventricle with kinetic abnormalities and decreased ejection fraction. The left ventricle was dilated as well. His father and the two brothers carrying the same missense mutation did not fulfilled the current diagnostic criteria for ARVC.

### p.I345T

The proband (II,1, Fam #170) (Figure 1b) was diagnosed at the age of 50 years, due to a sustained VT episode for which he received an implantable cardioverter defibrillator. The 12-lead ECG showed negative T wave in V4–V6, whereas late potentials were present at 40–80/250 filters. Two-D echocardiogram demonstrated a right ventricular enlargement with moderately depressed ejection fraction; the left ventricle was segmentary involved. The 15 years-old daughter, who did not show any arrhythmic symptom, was found to carry the same *DSC2 *mutation. The 2D-echocardiogram revealed mild right ventricular abnormalities, whereas 12-lead ECG was normal.

All family members not carrying *DSC2 *mutations were negative at clinical investigation.

### Functional Analysis of N-terminal Mutant Desmocollins

To evaluate the pathogenic potentials of the *DSC2 *missense mutations reported in the present study, full-length wild-type cDNA was directionally cloned in eukaryotic expression vector to obtain a fusion protein with GFP. Mutated proteins carrying p.E102K and p.I345T were obtained by site directed mutagenesis of the wild-type construct. Constructs were transfected in neonatal rat cardiomyocytes and in the desmosome-forming cell line HL-1.

**Figure 2:**
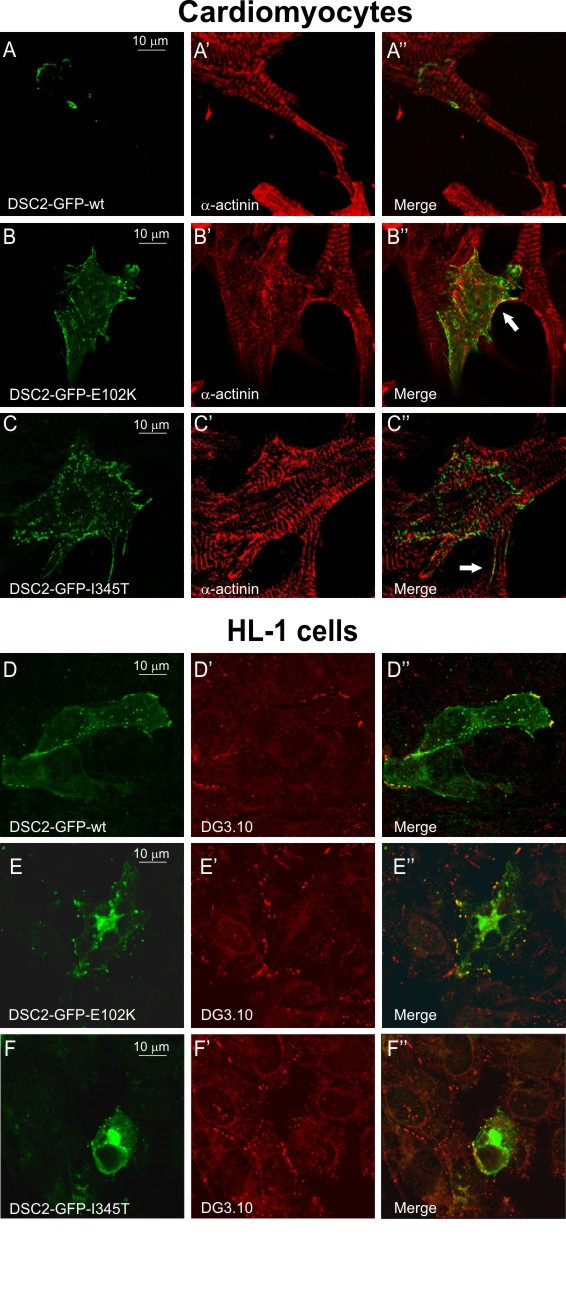
Transfection studies in cultured neonatal rat cardiomyocytes (top panels) and HL-1 cells (bottom panels). Note the WT-DSC2a-GFP was localised at the cell membrane between two cardiomyocytes and HL-1 cells (panel A and D), whereas E102K and I345T-DSC2a-GFP were mainly detected in the cytoplasm (panel B, C, E and F). As a reference, in cardiomyocytes immunostaining of the same cells with a monoclonal anti-alpha-actinin (panel A', B' and C') and overlay of the DSC2a-GFP and alpha-actinin staining (panel A", B" and C") are shown. In HL-1 cells immunostaining with monoclonal desmoglein antibody DG 3.10 showed both the presence of well-assembled desmosomes (panel D', E' and F') and the reduced co-localisation between endogenous dsg and mutated DSC2 (yellow dots in panel E", F").

In neonatal rat cardiomyocytes, wild-type fusion protein was localised at the cell membrane, only at the interface between two neighbouring cells (Figure 2, panel A). By contrast, protein carrying the p.E102K mutation was distributed in dots both in the membrane and in the cytoplasm (Figure 2, panel B), whereas protein carrying p.I345T mutation was predominantly localised in the cytoplasm (Figure 2, panel C). Moreover, the membrane localisation of both mutated proteins was not restricted to cell-cell junctions (see arrows in Figure 2 panel B" and C").

In transfected HL-1 cells, wild-type fusion protein was detected in the cell membrane, into cell-cell contact regions (Figure 2, panel D), and co-localised with the endogenous dsg, which was marked with monoclonal desmoglein antibody (Figure 2, panel D' and D"). This co-localisation suggests that the wild-type fusion protein has been integrated into normal-appearing desmosomes. p.E102K and p.I345T mutant fusion proteins showed the same distribution observed in neonatal rat cardiomyocytes (Figure 2, panel E and F).

In addition, immunostaining with monoclonal desmoglein antibody showed both the presence of well-assembled desmosomes in transfected HL-1 cells (Figure 2, panel E', F') and the reduced co-localisation between endogenous dsg and mutated DSC2 (Figure 2, panel E", F").

## Discussion

In the present study, we identified two mutations in *DSC2 *gene associated with ARVC, a genetically determined heart muscle disease characterized by structural, electrical, and pathological abnormalities of the right ventricle. Thus far, only 3 *DSC2 *mutations have been reported in ARVC patients [9,10]. On the basis of present data and of those previously reported [9,10], DSC2 mutations account for only a small percentage of ARVC probands.

Desmosomal cadherins, DSGs and DSCs, mediate cell-cell adhesion by interacting laterally and transcellularly with each other and by recruiting cytoplasmic plaque proteins which facilitate attachment of intermediate filaments. In particular, DSCs dimerise laterally via homophilic and heterophilic interactions at the cell surface and also make head-to-head contacts across the intercellular gap to form the so-called "adhesive zipper" [19]. Moreover, it has been suggested that DSC2 expressed at cell surface might act as nucleation centres for subsequent assembly of functional desmosomes [20].

Desmocollins bind desmosomal cadherins through their extracellular domains, while the cytoplasmic domains have binding sites for desmosomal plaque proteins. DSCs occur as "a" and "b" splice variants, with the "a" variant having a slightly longer cytoplasmic domain. It has been shown that the "a" variant can support desmosomal assembly [21], but there is no known function for the "b" form. In humans, there are three desmocollin isoforms (DSC1–3); DSC2 is almost ubiquitous in human tissues, but it is the only desmocollin isoform expressed in cardiac tissue [22].

DSC2 is a membrane glycoprotein composed of four highly conserved extracellular subdomains (EC1–4), a more variable extracellular anchor domain (EA), a single transmembrane domain (TM), an intracellular anchor domain (IA) and a cytoplasmic region that can show two different isoforms.

Cadherins are synthesized as inactive precursor proteins containing a prosequence followed by the cadherin domains. The N-terminal prosequence is proteolytically cleaved off in the late Golgi and the mature cadherin is then transported to the plasma membrane. Proteolytic removal of the prosequence results in structural rearrangements within EC1 domain with the activation of adhesive properties [23]. Mutation p.E102K alters a conserved amino acid located in the propeptide region.

Mutation p.I345T is located in the EC2 domain, which forms, together with EC1, the "minimal essential unit" to mediate cell adhesion, through *cis *and *trans *interactions among desmosomal cadherins [24].

In vitro functional studies on neonatal rat cardiomyocytes and on HL-1 cells demonstrate that the two missense mutations in the N-terminal domain affect the normal localisation of DSC2. As previously reported, the wild-type DSC2a-GFP fusion protein was efficiently incorporated into desmosomes and did not exert dominant-negative effect when overexpressed [25]. A lower amount of GFP signal was detected in the cytoplasm, since proteins were still not fully trafficked to the membrane. Unlike wild-type DSC2, the N-terminal mutants were predominantly located in the cytoplasm. Further investigation will be needed to determine whether the mutations could induce "null alleles" potentially leading to haploinsufficiency (i.e. through cytoplasmic degradation) or the mutant proteins could be present in the tissue and act in a dominant fashion (i.e. gain-of-function).

While the involvement of genes encoding desmosomal proteins in ARVC suggests that disruption of desmosomal integrity might be among primary molecular defects [8], pathogenetic mechanisms leading to ARVC remain to be elucidated. Recent data from Garcia-Gras et al. on cardiac restricted *dsp*-deficient mice would point at a novel molecular mechanism through suppression of Wnt/β-catenin signaling by nuclear plakoglobin [26].

*DSC2 *mutations were detected in two ARVC probands and in four family members who met only minor diagnostic criteria. This is consistent with incomplete penetrance of the disease, as previously reported in ARVC patients family members carrying *DSP*, *PKP2 *and *DSG2 *mutations [7,27-29]. However, due to the young age of most of the family members carrying the DSC2 mutations, we cannot exclude that some of them could later show clinical signs of the disease.

None of the probands carrying the pathogenic mutations showed gross skin/hair abnormalities. All DSC isoforms are expressed in the epidermal tissues, whereas *DSC2 *is the only one expressed in the myocardium. We may hypothesize that compensation by other DSC isoforms might take place in the epidermis but not in the myocardium of *DSC2 *mutation carriers, thus accounting for the cardiac-specific phenotype.

## Conclusion

In conclusion, we identified two novel missense mutations in *DSC2 *gene associated with ARVC. In vitro functional test demonstrated that N-terminal mutated *DSC2 *is almost exclusively distributed throughout the cytoplasm, thus suggesting the potential pathogenic effect of the reported mutations.

Identification of additional DSC2 mutations associated with ARVC may result in early detection of asymptomatic carriers and in increased diagnostic accuracy in the clinical evaluation of family members.

## Competing interests

The author(s) declare that they have no competing interests.

## Authors' contributions

GB performed the mutation screening of DSC2 gene in ARVC patients and carried out the in vitro functional studies. MDB, AL and LS set up DHPLC conditions and performed the mutation screening of DSC2 gene in ARVC patients. AN, BB, CB and GT were in charge of the clinical management of all patients. MS and GL performed the transfection experiments on HL-1 cells. MZ and LM performed transfection experiments on neonatal rat cardiomyocytes. GO designed the PCR primers and conditions used in the mutation screening. JT, head of the NIH project that partially funded our studies, organized the collaborative aspect of the project and provided a critical revision of the paper. GAD contributed to the original design of the study and actively participated in the writing of paper. AR supervised and coordinated the whole project and co-wrote the paper with GB.

All authors read and approved the final manuscript.

## Pre-publication history

The pre-publication history for this paper can be accessed here:


